# 3D-Printed Biosensor Arrays for Medical Diagnostics

**DOI:** 10.3390/mi9080394

**Published:** 2018-08-07

**Authors:** Mohamed Sharafeldin, Abby Jones, James F. Rusling

**Affiliations:** 1 Department of Chemistry (U-3060), University of Connecticut, 55 North Eagleville Road, Storrs, CT 06269, USA; mohamed.sharafeldin@uconn.edu (M.S.); abby.jones@uconn.edu (A.J.); 2 Analytical Chemistry Department, Faculty of Pharmacy, Zagazig University, Zagazig 44519, Sharkia, Egypt; 3 Institute of Materials Science, University of Connecticut, 97 North Eagleville Road, Storrs, CT 06269, USA; 4 Department of Surgery and Neag Cancer Center, UConn Health, Farmington, CT 06032, USA; 5 School of Chemistry, National University of Ireland, Galway, University Road, Galway, Ireland

**Keywords:** 3D printing, diagnostics, optics, bioprinting, electronics, microfluidics

## Abstract

While the technology is relatively new, low-cost 3D printing has impacted many aspects of human life. 3D printers are being used as manufacturing tools for a wide variety of devices in a spectrum of applications ranging from diagnosis to implants to external prostheses. The ease of use, availability of 3D-design software and low cost has made 3D printing an accessible manufacturing and fabrication tool in many bioanalytical research laboratories. 3D printers can print materials with varying density, optical character, strength and chemical properties that provide the user with a vast array of strategic options. In this review, we focus on applications in biomedical diagnostics and how this revolutionary technique is facilitating the development of low-cost, sensitive, and often geometrically complex tools. 3D printing in the fabrication of microfluidics, supporting equipment, and optical and electronic components of diagnostic devices is presented. Emerging diagnostics systems using 3D bioprinting as a tool to incorporate living cells or biomaterials into 3D printing is also reviewed.

## 1. Introduction

Charles W. Hull in 1986 was the first to report stereolithography [1] as a tool to fabricate 3D structures. Since then, 3D printing has evolved into a multifunctional fabrication tool that offers unique advantages for biomedical applications including diagnostics [2], scaffolds for 3D implants [3], prosthesis [4] and tissue engineering [5]. In recent years, the ability to convert computer-assisted design (CAD) files into 3D-printed pieces, also known as additive manufacturing, has sparked significant progress in the field of diagnostics [6]. 3D printing has been utilized in a wide spectrum of applications with excellent design and performance. As an additive manufacturing technique, production costs are lower compared to traditional subtractive manufacturing techniques like milling or ablation due to reduction of the labor and material cost. In addition, versatile 3D printers can be used to produce different devices and parts without the need for pre-fabrication changes normally required in subtractive manufacturing techniques [7,8]. These criteria make 3D printing a valuable tool in prototyping, testing and production of tools and equipment for analytical and diagnostic laboratories. In principle, CAD files of previously reported devices can be downloaded and printed in any laboratory. In this way, advanced diagnostic tools can be directly utilized by researchers without the need for purchase from a commercial vendor. This approach has the potential to bring advanced diagnostic tools more rapidly to the research lab than ever before. 

In this review, we focus on applications of 3D printing techniques in medical diagnostics. We discuss different 3D printing techniques and how these techniques impact many design aspects including resolution, cost and fabrication of complex diagnostic devices in a continuous process [9,10,11]. 3D-printed microfluidic devices have been used to fabricate semi and fully automated diagnostic approaches for diseases like cancer [12,13], infectious diseases [14,15,16], and xenobiotic genotoxicity [17]. 3D printing can also make tailored supporting devices that improve performance of existing diagnostics like spectrophotometers [18] and Polymerase Chain Reaction (PCR) devices [14,19] and is used to assist with smartphone integration for remote sensing [20,21]. The ability to print materials with special properties allows for the creation of new equipment that can dramatically reduce the cost of diagnostic devices like Surface Plasmon Resonance (SPR) [22]. All these applications use 3D printing for cost-effective multifunctional production to integrate several functions in one device [23]. 

Fabrication of diagnostic devices with embedded electronics and circuits have also been accomplished by 3D printing. The ability to print different materials simultaneously permits the fabrication of electrodes that can be incorporated into the insulator plastic matrices allowing for subsequent electrochemical detection of metals [24,25,26], organic compounds [27,28] and biologically active molecules [29]. 3D printing avoids disadvantages associated with screen printing like the need for masking and drying steps, while exhibiting better resolution and faster fabrication [30]. 

3D bioprinting is another emerging modification to traditional 3D printing where cells, enzymes or proteins may be encapsulated or loaded into printable bio-ink solutions [31]. A major focus of this technique is to provide cell growth medium for tissue and organ repair and regeneration, but it has also been explored as a tool for diagnostic applications [32]. Bioprinting offers an opportunity to fabricate 3D-printed implantable sensors that are biocompatible, geometrically complex, and cheap. With 3D printing, there is a limited need for specialized training, and devices can be tailored to the users’ requirements [33,34]. In this review, the most common techniques for 3D-printed diagnostics are briefly described with several examples of diagnostic platforms incorporating microfluidics, device supports, optical components, electronics and biomaterials. 

## 2. Additive Manufacturing Techniques

### 2.1. Fused Deposition Modeling (FDM)

**Figure 1:**
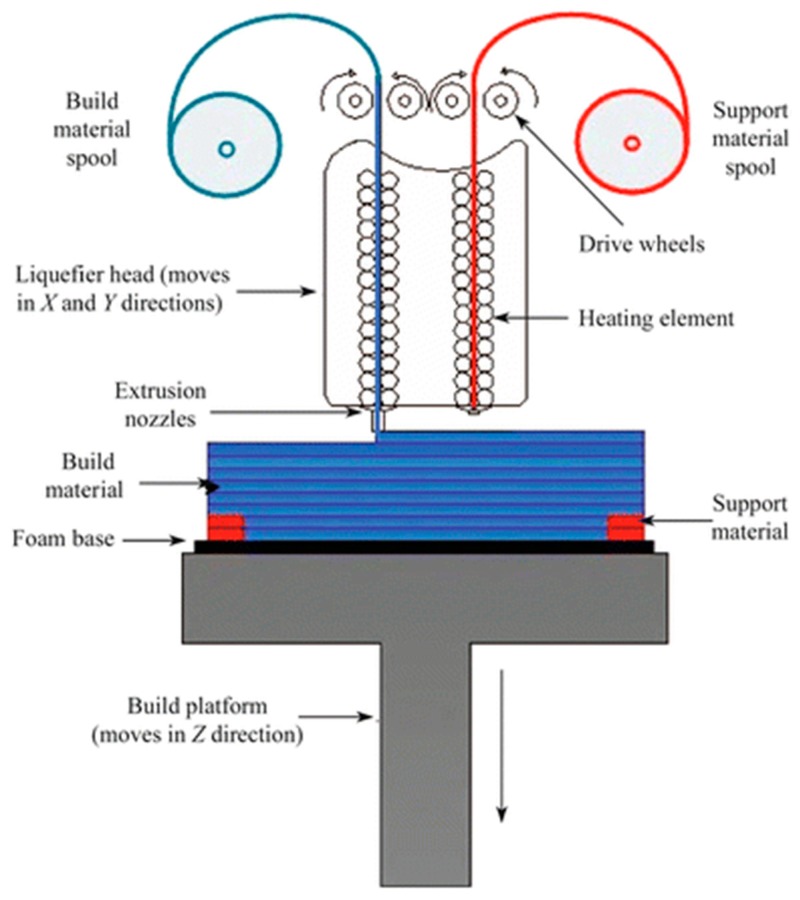
Schematic representation of a dual-head fused deposition modeling 3D printer. Thermoplastic polymer is extruded from a heated nozzle into a printing platform, where it is cooled to below its thermoplastic temperature. Reproduced with permission from [35]. Copyright (2015) Springer.

This technique utilizes thermoplastic polymeric materials extruded to print objects layer-by-layer from a heated nozzle onto a surface or platform where it is cooled to below its thermoplastic temperature (Figure 1). Several materials have been utilized in this printing technique, including acrylonitrile butadiene styrene (ABS), polycarbonate (PC), PC-ABS blend, and polylactic acid (PLA) [35]. Single-, double- and triple-print-head machines are available for FDM, making it a good choice for simultaneous multi-material 3D printing [36]. The ability to incorporate conductive materials like pyrolytic graphite, graphene, carbon nanotubes and metal nanoparticles into the thermoplastic matrix enables FDM printing of conductive inks to fabricate electrodes and circuits [37,38,39,40]. FDM is good for rapid prototyping and fabrication of holders and supporting devices, but still suffers from several limitations, including mechanical strength, roughness and shape integrity of the final product. Microfluidic devices printed using FDM can show leakage and shape deformation if printing parameters and the thermoplastic polymer are not carefully tuned [37]. FDM has been successfully used to print 3D scaffolds that can be seeded with living cells without loss of cell viability [41,42] and to print bio-friendly polymer materials [43,44].

### 2.2. Direct Ink Writing

Similar to fused deposition modeling, Direct Ink Writing (DIW) relies on extrusion of ink through a fine deposition nozzle to form a 3D structure in layer-by-layer approach [45]. Two different strategies are utilized in this technique, based on the ink type. First is the extrusion of low-viscosity ink that undergoes gelation via a chemical, photochemical or noncovalent process [46]. The second strategy is the use of a shear thinning hydrogel ink that possesses a viscoelastic response toward applied pressure. Hydrogels like sodium alginate and gelatin are commonly used [47]. In addition to hydrogel inks, epoxy-based direct writing was developed by Compton and Lewis [48], where epoxy ink with significant shear thinning is extruded through the printing nozzle. Once extruded, the ink has sufficient shear to maintain its printed filamentary shape. Direct ink writing is utilized in the 3D fabrication of injectable therapeutics [49], cell-laden scaffolds [50,51], degradable biomaterials [52] and stretchable complex cellularized structures [53]. The utilization of DIW in bioprinting offers a tool to develop multifunctional diagnostic devices with high resolution, which may improve assay sensitivities. 

### 2.3. Stereolithography

**Figure 2:**
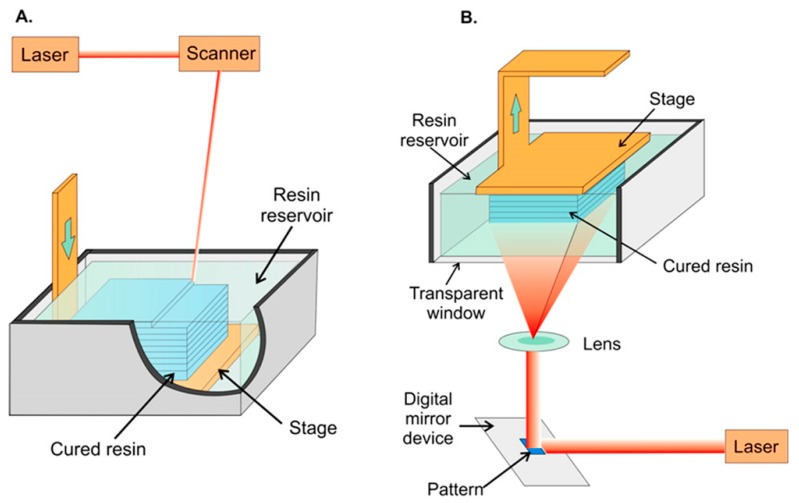
Schematic representation of stereolithographic 3D printing. (**A**) Scanning laser stereolithography, where the focused laser beam scans point-by-point to cure a layer of resin on top of a previously fabricated layer. (**B**) Projection-based stereolithography, where an entire layer is printed in a single step by projecting the entire layer on top of the previous layer. In both strategies, a printing platform is immersed in a tank filled with liquid photocurable resin. Reproduced with permission from [54]. Copyright (2014) American Chemical Society.

Stereolithography, or digital light processing, employs a photocurable polymeric resin which, when exposed to light, cures into a solid. Initially, curing was only possible with UV light, but polymers cured with visible wavelengths have recently been introduced. Highly focused lasers or LED beams with high intensity are used and the spot size of the light beam determines printing resolution [54]. Each layer of the object is printed as a point-by-point 2D cross section cured by the scanning focused beam onto a printing platform immersed in a photocurable tank that holds the liquid resin [5] (Figure 2A). Recently, projection-based stereolithography has been introduced with a promise to decrease print time while maintaining almost the same resolution as line-based stereolithography. Projection-based stereolithography replaces point-by-point curing with entire-layer curing under one single UV or visible light exposure [55,56] (Figure 2B). Stereolithography resin materials have been extensively studied to produce devices with different properties, including transparency, color, flexibility and thermal stability [57]. Stereolithography has also been used for printing cells using biocompatible resins maintaining >90% cell viability after printing [58]. 

### 2.4. Photopolymer Inkjet Printing (Multi-Jet Modeling—MJM)

**Figure 3:**
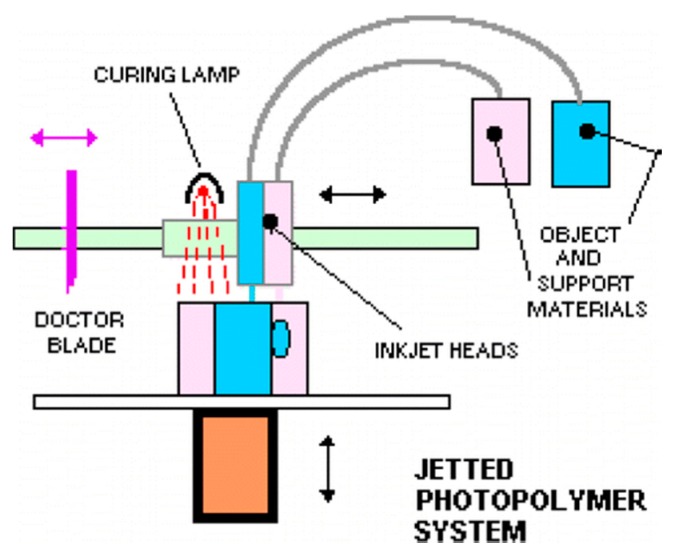
Schematic representation of multi-jet printing technique, a photocurable resin is printed simultaneously with a support material that can be removed after curing. Up to 10 printing heads can be used. Reproduced with permission from [59]. Copyright (2014) American Chemical Society.

This technique utilizes multi-head printers with print heads similar to inkjet printers. The print head extrudes layers of photocurable resin or molten wax, usually with a second head printing support material to maintain the shape of the design until cured (Figure 3). After printing, the object is cured by UV irradiation or heat and support material can be removed by heating or dissolving in a specific solvent [59]. Researchers have been able to utilize this printing technique to print metal nanoparticles for printed electronics [60], preceramic polymers for 3D-printed ceramics [61] and even metallic electrodes on flexible substrates [62]. The ability to print multiple materials with varying chemical and physical properties simultaneously makes MJM a good candidate for diagnostic device fabrication. Microfluidic channels integrated with either electrodes for electrochemical signal detection [63] or porous membranes that can be seeded with viable cells for drug permeability and toxicity studies have been printed using this technique [64]. Most printing resins and materials are proprietary, which makes the cost of using MJM relatively higher than other 3D-printing techniques [65].

### 2.5. Selective Laser Sintering (SLS)

**Figure 4:**
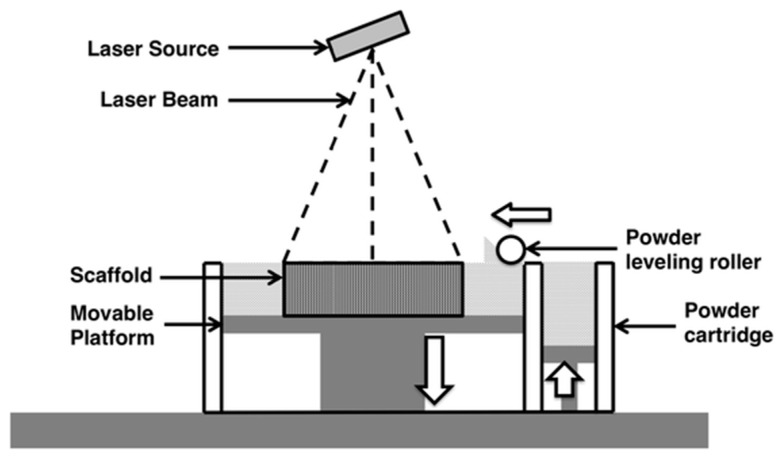
Schematic representation of Selective Laser Sintering, a rolling ball pushes powdered substrate to the surface of the printing platform. A high-energy focused laser beam scans the surface where it sinters the powder particles into a solid layer. The printing chamber is sealed under vacuum or inert gas atmosphere. Reproduced with permission from [70]. Copyright (2015) Springer Nature available under Creative Commons Attribution.

A focused Infra-Red (IR) laser beam supplies enough localized energy to sinter a fine powdered polymer into layers of solid. The IR laser scans through the surface of powder in the shape of each layer of the sliced 3D design (Figure 4). Due to the high energy required to sinter powders, high-energy CO2/Nd:YAG laser sources are typically used [66]. SLS can be divided into two distinctive subcategories based on the printing temperature. The first is solid-state sintering, where binding occurs at a temperature lower than the melting temperature and is usually used with polymers like polycarbonate. The second is full melting SLS and is used for metals and ceramics where sintering requires a high temperature above the melting temperature [67]. Printing resolution is affected by powder particle size and can be controlled by the scan speed and intensity of the laser beam, which also affects the density and strength of the printed parts [68]. SLS has utilized several printing substrates, including natural and synthesized polymers like cellulose and polycarbonate, making it compatible with bioprinting for tissue engineering and cartilage repair [69]. Other printing substrates include metals, ceramics and polymer/ceramic composites. It is important to note that the printing resolution of polymers is much lower compared to that of metals or ceramics [70]. Due to the high-energy laser source required and the substrate specifications, SLS is currently considered to be the most expensive 3D printing technique [71]. Recently, Formlabs introduced Fuse1, a desktop SLS printer that provides end-users with a more affordable option [72].

### 2.6. Direct Laser Writing (DLW) 3D Lithography

Direct laser writing is an emerging technique for 3D printing of high-resolution structures utilizing a highly pulsed femtosecond laser beam to cure a photosensitive resin material [73]. Two-photon absorption and polymerization facilitates fast fabrication of 3D scaffolds with high resolution [74]. This short pulsed laser is suitable for encapsulating living cells and biomaterials in 3D structures as it does not generate localized overheating or UV toxicity. Due to versatility in substrate materials and the ability to print high-resolution 3D scaffolds, DLW has been used in piezoelectric scaffolds for in vitro cell stimulation [74], cartilage tissue engineering [75] and cells and whole organisms containing 3D structures [76,77,78]. Due to the high resolution achieved by DLW, it has been utilized in fabrication of microvalve assembly [79], custom microstructures [80,81] and complex microfluidic constructs [82,83].

### 2.7. Summary of 3D Printing Techniques

In order to select the appropriate 3D printing technique, the user must have in mind the properties required for the printed piece. Several criteria, such as flexibility, resolution, complexity, transparency, thermal and chemical stability, are crucial in determining the best technique. Table 1 summarizes the 3D printing techniques discussed in this section.

## 3. Applications of 3D Printing in Diagnostics

3D printing has improved biomedical diagnostics in many ways, specifically with advantages in ease of onsite design and fabrication, providing researchers with the means to develop or modify devices and equipment. Here we concentrated on the main areas in which biomedical diagnostic research has been focused recently.

### 3.1. 3D-Printed Microfluidics

The most representative use of 3D printing technology in diagnostics is the design and development of microfluidic devices. The ability to fine-tune geometrically complex structures at the micrometer level is an attractive feature 3D printing can offer while maintaining low-cost and time-efficient processing. Several applications that have used 3D-printed microfluidic devices are discussed.

#### 3.1.1. Sample Pretreatment

**Figure 5:**
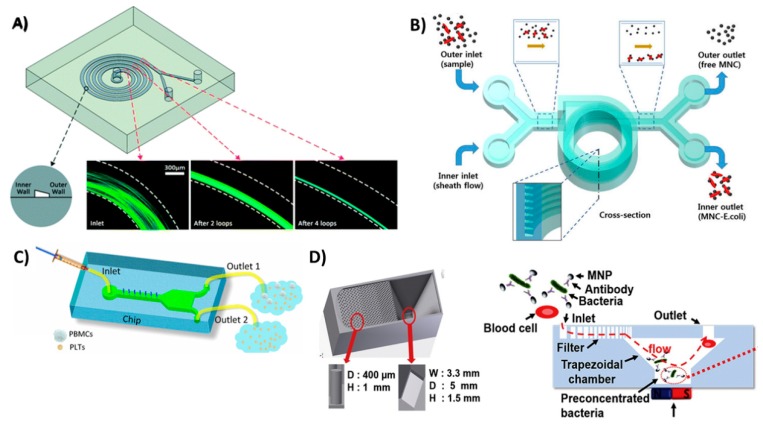
3D-printed devices for sample pretreatment. (**A**) Spiral microfluidic device to separate blood cells and platelets from plasma, as the cells and platelets tend to flow in a narrowing band near the inner wall of the spiral channel. Reproduced with permission from [88]. Copyright (2016) Royal Society of Chemistry. (**B**) Helical microfluidic device to separate magnetic nanoclusters coupled to *E. coli* from free magnetic nanoclusters. Reproduced with permission from [89]. Copyright (2015) Springer Nature, available under Creative Commons Attribution. (**C**) A hand-driven microfluidic channel with a groove-like structure to separate platelets from blood mononuclear cells. Reproduced with permission from [90] Copyright (2018) Springer Nature. (**D**) Trapezoidal filter equipped with a microfluidic channel for the preconcentration of *E. coli* captured on magnetic beads. Reproduced with permission from [91] Copyright (2017) Elsevier.

Sample pretreatment is an essential step in many diagnostics, as it helps reduce the complexity of the matrix and improve the sensitivity of the assay. 3D-printed microfluidics facilitates the integration of sample pretreatment compartments into real applications including sample injection valves [84,85], preconcentration [86] and sample reactors [87]. Rafeie et al. utilized 3D printing to fabricate an ultrafast microfluidic blood plasma separator, an essential sample pretreatment step in most assays requiring blood samples. They were able to fabricate a spiral microfluidic device (Figure 5A) where cells would flow close to the inner wall of the channel and concentrate in a narrow band near the outlet allowing the separation of cell/platelet free plasma. [88]. Lee et al. separated pathogenic bacteria, *E. coli*, from milk using a 3D-printed helical channel [89]. They flowed magnetic nanoclusters through the helical microfluidic channel (Figure 5B) where free magnetic nanoclusters were separated from bacteria-bound clusters. Yan et al. proposed a portable hand operated microfluidic device that can specifically separate platelets from peripheral blood mononuclear cells [90]. Their device is composed of a microfluidic channel equipped with a groove (Figure 5C) that effectively sorts platelets from blood samples with 100% purity where the user pumps the fluid manually with a hand-held syringe. While fluctuation in the flow rate did not affect the platelet purity, the percent recovery of blood mononuclear cells varied. A microfluidic pre-concentrator for detection of *E. coli* was also proposed by Park et al. [91]. Magnetic nanoparticles labeled with *E. coli*-specific antibodies were allowed to capture bacteria from blood samples. The microfluidic device was equipped with a magnet to separate (Figure 5D) magnetic nanoparticles from the blood matrix which then transferred with buffer for adenosine triphosphate (ATP) luminescence analysis. Although these devices are interesting applications for 3D printing in sample pretreatment, they still require a manual transfer of the treated samples for detection. This manual transfer can negatively affect the assays sensitivity and reproducibility required for a good diagnostic approach.

#### 3.1.2. Microfluidic Flow Devices

**Figure 6:**
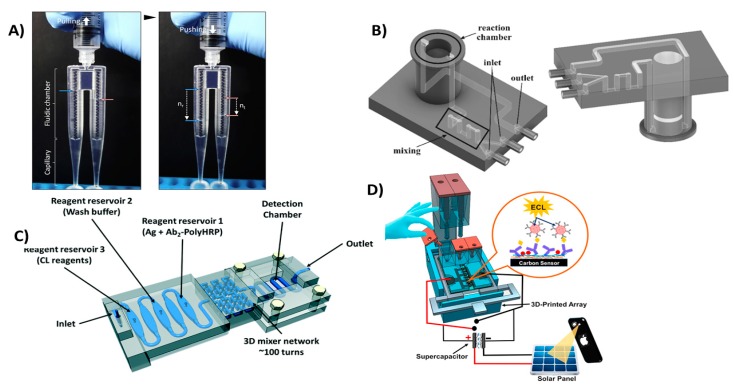
3D-printed microfluidic devices for flow control. (**A**) Viscometer like 3D-printed syringe attachment for blood viscosity measurement. Reproduced with permission from [92]. Copyright (2018) Elsevier. (**B**) Mixing and detection microfluidic device for luminescent detection of ATP. Reproduced with permission from [93]. Copyright (2018) Elsevier. (**C**) Unibody 3D-printed microfluidic chip for detection of PSA and PF-4. Reproduced with permission from [94]. Copyright (2017) Royal Society of Chemistry. (**D**) Manually controlled flow regulatory system for electrochemiluminescence detection of PSA, PSMA and PF-4. Reproduced with permission from [12]. Copyright (2016) Elsevier.

Microfluidic devices offer the most promising approach for miniature fluidic devices due to their ability to handle small sample volumes and assay reagents in a controlled manner. 3D printing has pushed prototyping and development of microfluidics forward by supporting fast and easy design with lower production costs compared to traditional microfabrication techniques. 3D printing also offers an efficient tool to generate geometrically complex microfluidic devices with the aid of 3D design software, thus eliminating the hassle associated with traditional manufacturing tools. Utilizing these advantages, Oh et al. designed and fabricated a 3D-printed blood viscosity analysis capillary circuit [92]. They designed a hand-held device that can be operated and read manually that measures blood viscosity using the same principle as commercial viscometers which are very expensive and complex (Figure 6A). Surprisingly, their device did not utilize the resolution advances of 3D printing, but instead they added Tygon tubing, with inner diameter of 0.508 mm, to build a capillary circuit inside a 3D-printed channel. Santangelo et al. proposed a highly sensitive 3D-printed continuous-flow microfluidic device for quantification of adenosine triphosphate (ATP) molecules (Figure 6B). The device comprised two main functions: mixing of the ATP sample with the luminescence reagent mixture (Luciferin/Luciferase mixture) and a detection chamber that brings the produced luminescence close to a silicon photomultiplier detector [93]. Tang et al. utilized 3D printing to fabricate a unibody ELISA-inspired chemiluminescence assay to detect and quantify prostate specific antigen (PSA) and platelet factor-4 (PF-4) as cancer biomarker proteins (Figure 6C) [94]. They proposed a design that can reduce the assay time to 30 min while approaching an ultra-low sensitivity. Their design is divided into three connected compartments: first, a mixing chamber to accelerate the interaction between reagents; second, a compartment of sample and reagent reservoirs; and third, a transparent detection compartment. The ability to 3D print transparent objects allowed them to directly detect the chemiluminescent signal in their device using a CCD camera without the need for complex processing. Recently, a Lego-like modular microfluidic capillary-driven 3D-printed flow device was introduced by Nie et al. [95]. This approach proposed a strategy to build microfluidic devices tailored to different applications. Flow in such devices is driven by capillary forces, with improved flow rate programmability and biocompatibility. They were able to design different modules assembled in various designs and utilized them in diverse applications, like degradable bone scaffolds and cell culture. Kadimisetty et al. proposed a 3D-printed microfluidic unit that manually controls the flow of sample and assay reagents for electrochemiluminescent detection of PSA, PF-4 and prostate specific membrane antigen (PSMA) in human serum [12]. The printed device had a slot to incorporate a screen-printed carbon electrode labeled with detection antibodies for each of the selected protein biomarkers (Figure 6D). Similar 3D-printed microfluidic flow devices were fabricated and utilized for flow chemical analysis [96], evaluation of blood components [97], electrochemiluminescence DNA studies [98] and salivary cortisol detection [99]. In these discussed examples, 3D printing was the key for better diagnostic performance by providing low-cost incorporation of multiple fluidic functions easily and without the need for laborious manufacturing procedures. 

#### 3.1.3. Microfluidic Mixers

**Figure 7:**
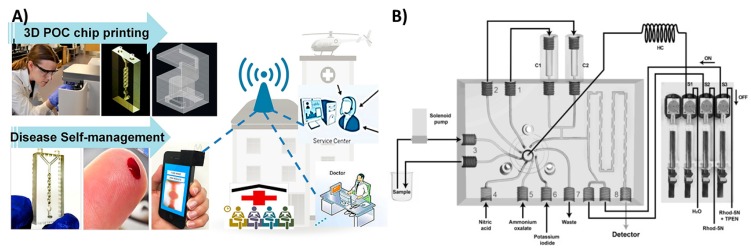
3D-printed microfluidic mixers. (**A**) Microfluidic mixer for tele-diagnosis of anemia. Less than one second of mixing required for the blood sample with the oxidizing agent; generated colorimetric signal detected with a smartphone. Reproduced with permission from [105]. Copyright (2016) AIP Publishing. (**B**) A lab on valve complex 3D-printed microfluidic chip for quantification of lead and cadmium in water samples. Reproduced with permission from [106]. Copyright (2018) Elsevier.

Efficient mixing can be used to improve diagnostic tests by enhancing interaction kinetics between reactants. Microfluidics can be configured for efficient mixing to enhance chaotic convection in solutions, increasing the frequency of interactions between solution components [100]. 3D-printed microfluidic mixers have been successfully used to improve passive mixing enhancing mixing efficiency that improve diagnostics sensitivity [101]. Devices equipped with 3D-printed mixers have been used in amperometric quantitation of hydrogen peroxide [102] and DNA assembly [103]. 3D-printed mixers have been successfully integrated with optical spectroscopic probes including UV/Vis, infrared and fluorescence probes [104]. Plevniak et al. proposed a 3D-printed microfluidic mixer for diagnosis of anemia (Figure 7A). In their work, they were able to integrate the device with smartphone-aided colorimetric signal detection to overcome the distance barrier for efficient screening [105]. The device can analyze a finger prick of blood (~5 µL) driven by capillary force into the mixing chamber where it is mixed with an oxidizing agent in less than 1 sec with cost 50 cents/chip. Another mixing device was introduced by Mattio et al. [106], where a complex valve design was fabricated using 3D printing (Figure 7B). The device has eight inlets for sample and reagents connected to a valve where samples and reagents are mixed and transferred to detector. The device was used to quantify Lead and Cadmium in water samples extracted from soil. One inlet was used for nitric acid required for column conditioning, other inlets were used for fluorescence reagent, Rhod-5N™, and co-reagents potassium iodide, N,N,N′,N′-tetrakis-(2-Pyridylmethyl)ethylenediamine (TPEN) and ammonium oxalate.

#### 3.1.4. Multifunctional Microfluidics 

**Figure 8:**
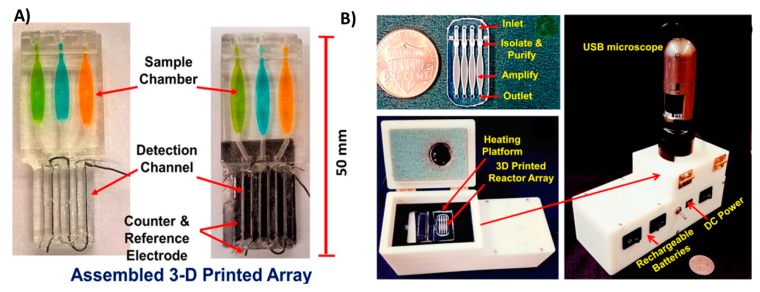
Multifunctional 3D-printed microfluidics. (**A**) A 3D-printed chip to detect genotoxicity of metabolites from e-cigarette extracts. The device has a sample and reagent reservoir compartment and a detection compartment equipped with platinum counter electrode and Ag/AgCl reference electrode. Reproduced with permission from [17]. Copyright (2017) American Chemical Society. (**B**) A 3D-printed microfluidic array for isolation of nucleic acids equipped with a separation membrane and heating compartment to amplify nucleic acids using loop mediated isothermal amplification that can be attached to a USB microscope for fluorescence detection. Reproduced with permission from [9]. Copyright (2018) Elsevier.

In the previous examples, 3D printing was utilized to fabricate microfluidics that served only one purpose. Several researchers have proposed multifunctional 3D-printed microfluidic devices capable of performing several tasks simultaneously. Kadimisetty et al. introduced a microfluidic device that can analyze extracts from e-cigarette vapors [17]. The device is equipped with sample and reagent reservoirs, in addition to an electrochemiluminescence signal detection compartment (Figure 8A). Another multifunctional microfluidic device was also introduced recently by Kadimisetty et al. [9], where they were able to extract, concentrate and isothermally amplify nucleic acids in different bodily fluids as an approach for microfluidic point of care diagnostics (Figure 8B). The microfluidic device is integrated with a membrane to isolate nucleic acids, then placed in a chamber where loop mediated isothermal amplification is induced. Finally, the signal is either detected colorimetrically by a mobile phone or by fluorescence with a portable USB fluorescence microscope. This demonstrates the promising utility of 3D-printed microfluidic devices in Point-of-care (POC) applications. Other multifunctional microfluidic devices have been proposed to detect Human immunodeficiency virus (HIV) antibodies [107], zika virus [108] and glucose [109].

### 3.2. 3D-Printed Sensing Electronics 

**Figure 9:**
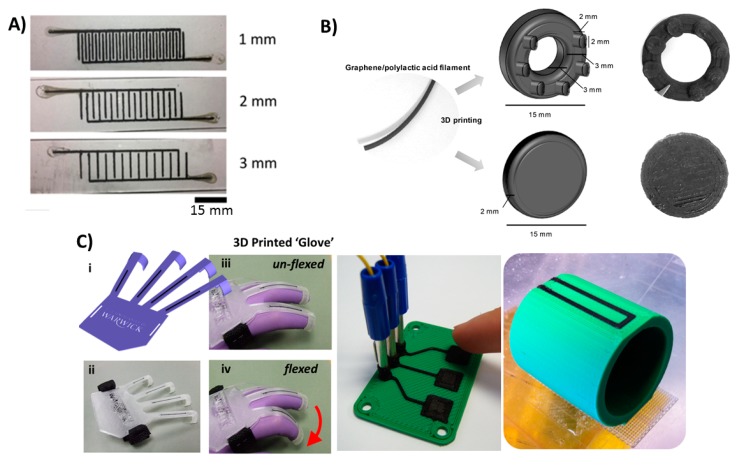
3D-printed electronics. (**A**) 3D-printed tactile electrode sensor. Conductive PDMS doped with carbon nanotubes were printed on PDMS or EcoflexTM to fabricate flexible electrode sensors. Reproduced with permission from [110]. Copyright (2018) IOP Publishing. (**B**) A 3D-printed graphene/polylactic acid electrode with ring or disc shape. Reproduced with permission from [111]. Copyright (2018) American Chemical Society. (**C**) A 3D-printed conductive carbon black electrode in different objects from left to right: flexible glove sensor, capacitive buttons and smart vessel. Reproduced from [38]. Copyright (2012) PLOS available under Creative Commons Attribution.

A number of researchers, especially those employing electrochemistry, are interested in 3D printing for its ability to design and fabricate sensing electronics. Supported by the versatility of printable materials, 3D printers have the ability to produce well defined shapes without masking required in traditional screen printing or photolithography This enables 3D printing to fabricate integrated electrode biosensors and electronic sensors that can be utilized as personal diagnostics devices and POC sensors. Li et al. used a home-made 3D printer to print a conductive polymer in polydimethylsiloxane (PDMS) or EcoflexTM to fabricate stretchable electrode sensors [110] (Figure 9A). Using this 3D printer, they achieved a resolution of 400 µm with an electrode height of 1 mm and detected sodium chloride electrochemically with a 1 μM detection limit and good sensitivity and reproducibility. Another approach for 3D printing electrodes using fused deposition modeling was proposed by Palenzuela et al. [111]. A commercially available graphene/polylactic acid filament was used to print electrodes of distinctive shapes designed on CAD software (Figure 9B). The printed electrodes were characterized using different redox probes and utilized to detect picric acid and ascorbic acid in solution. In order to fabricate more complex electronics, Leigh et al. used a triple-head fused deposition modeling printer to impede conductive filament within a nonconductive ABS or PLA matrix [38]. Using this approach, they were able to fabricate a variety of complex functional objects like a 3D flex sensor, capacitive buttons and a smart vessel (Figure 9C). The ability of 3D printing to develop electronic biosensing devices was demonstrated in the fabrication of strain sensors in biological systems [112,113] and skin-like sensors using thermo-responsive hydrogels [114]. Most of these applications are directed towards the fabrication of electronic skin, a promising diagnostic tool that composed of flexible and stretchable sensor that can perform several health monitoring functions like temperature, glucose, sodium chloride and pressure sensing [115]. 

### 3.3. 3D-Printed Supporting Devices

**Figure 10:**
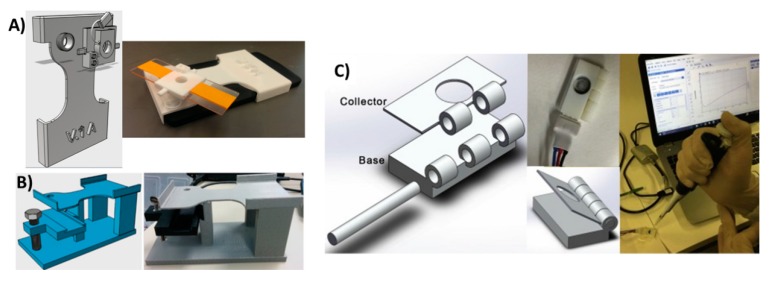
3D-printed support devices. (**A**) Soil analysis system with 3D-printed mobile phone holder equipped with a glass slide holder where samples were fixed in a lens in between the mobile camera and the sample holder. This jig has a replaceable filter just above the lens for fluorescence imaging. Reproduced from [115]. Copyright (2018) PLOS available under Creative Commons Attribution. (**B**) Alternate soil analysis system with the same support components, but modified to hold a microfluidic chip for flowing samples. Reproduced from [115]. Copyright (2018) PLOS available under Creative Commons Attribution. (**C**) Support device with sample application hole for paper-based electrochemical detection of butyrylcholinesterase activity. Reproduced with permission from [116]. Copyright (2018) Elsevier.

Versatility, ease of design and modification in a fast and economic manner made 3D printing the method of choice to develop the supporting equipment and pieces required for diagnostics. Shanmugam et al. used 3D printing to fabricate a custom designed mobile phone microscopy support unit. This unit perfectly aligns the sample compartment with simple optics and a mobile phone camera (Figure 10A) [116]. They also proposed a holder that can incorporate a microfluidic chamber for analyzing flowing samples rather than stationary samples (Figure 10B). Using such equipment, they were able to perform screening of soil-transmitted parasitic worms in resource-limited areas. Another supporting device for a paper-based electrochemical sensor was proposed by Scordo et al. [117]. A reagent-free sensor was proposed to test butyrylcholinesterase activity by detecting thiocholine. A 3D-printed support equipped with a sample application hole was used to provide the supporting strength and insulation required for electric connections (Figure 10C). Several 3D-printed supports for mobile phone-assisted diagnostics have been developed [118,119,120], in addition to equipment pieces that can lower the cost of current diagnostic strategies [19,121,122] without compromising performance.

### 3.4. 3D-Printed Optics

**Figure 11:**
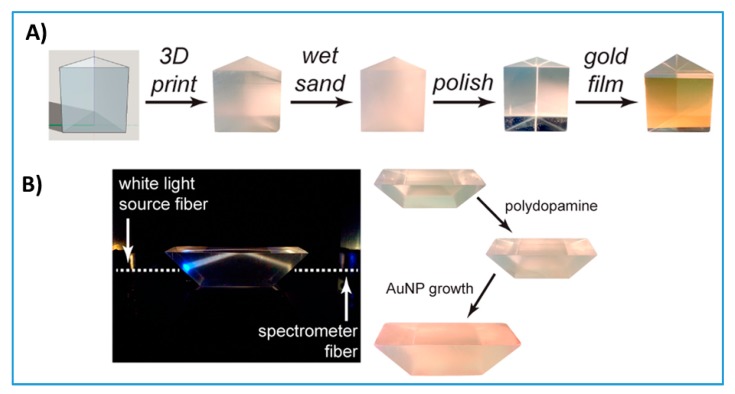
3D-printed optics. (**A**) 3D-printed prism polished with simple benchtop polishing decorated with a layer of gold and used for plasmonic sensing of cholera toxins. Reproduced with permission from [22]. Copyright (2017) American Chemical Society. (**B**) 3D-printed prism with a different geometry than (A) used to monitor nanoparticle growth. Reproduced with permission from [22]. Copyright (2017) American Chemical Society.

Despite the current limitations of the 3D printing of fully transparent surfaces without defects that could affect light reflection and transmission, researchers have tried to print functional optical components to reduce the cost and improve the performance of diagnostic devices. An interesting example from Hinamn et al. describes a 3D-printed prism that can be used for plasmonic sensing [22] (Figure 11A). In order to prove functionality, they deposited a layer of gold on one side of the prism and used it to detect cholera toxins. They also printed prisms with different geometries and used them to monitor nanoparticle growth (Figure 11B). Other researchers used two-photon polymerization 3D printing to fabricate high-resolution micro-optic components of optic fiber ends [123] and other micro-optics [124]. 3D-printed optical tweezers for sample trapping [125] were also developed to aid chemical and spectroscopic sample analysis. Some other 3D-printed fine optics that have not been used yet in diagnostics have also been proposed [126,127]. Integrating compact, lost-cost, effective optical components is a crucial development for POC diagnostics. These examples are good candidates for integration in POC optical diagnostic systems for medical testing [128]. 

3.5. 3D Bioprinting

**Figure 12:**
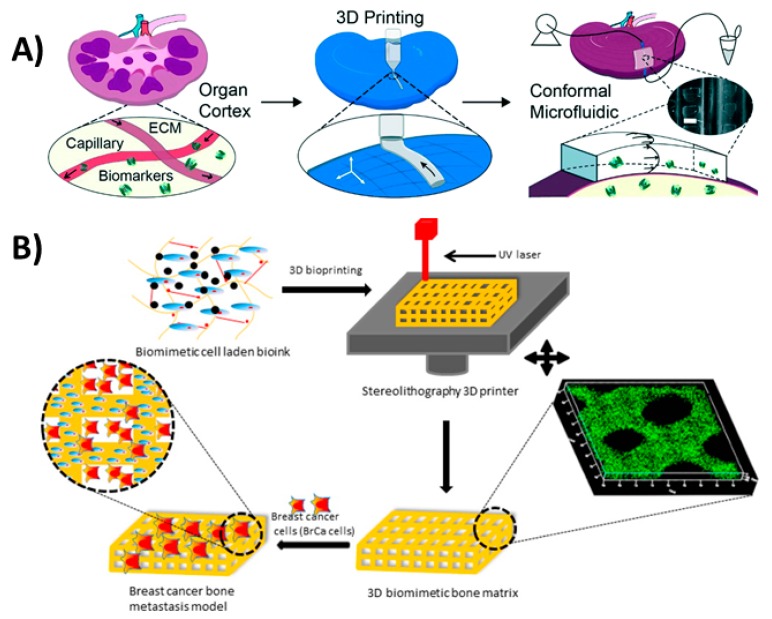
3D bioprinting. (**A**) 3D-printed perfusion chip for extraction of metabolites and biomarkers from whole organs. Reproduced with permission from [129]. Copyright (2017) Royal Society of Chemistry (**B**) 3D-printed bone-like scaffold carrying bone stromal cells to study their interactions with breast cancer cells. Reproduced with permission from [34]. Copyright (2016) American Chemical Society.

The ability to use biocompatible 3D printing substrates allowed the incorporation of biomaterials in 3D-printed scaffolds. This facilitated the further investigation of multifunctional 3D-printed devices that could express biomimetic activity in diagnostic applications. A bioinspired microfluidic chip that can be attached to a whole organ was proposed by Singh et al. [129]. This microfluidic chip was fabricated based on structured light scanning of a whole organ followed by stereolithographic 3D printing using the scanned conformation. The as-printed device was attached to porcine kidney for biomarker extraction and profiling (Figure 12A) without the need for tissue removal. This approach enables the study of metabolic activities in a living whole organ, paving the way for further investigation into drug toxicity screening and biomarker discovery.

A cell-laden bone matrix was proposed by Zhou et al. [34] to study breast cancer metastasis. They printed a gelatin-based methacrylate hydrogel with incorporated bone stromal cells to study their interactions with breast cancer cells (Figure 12B). An in vivo alkaline phosphatase testing platform was introduced by Park et al. [32] using 3D-printed biocompatible calcium-deficient hydroxyapatite. Although 3D bioprinting developments are mainly used in tissue constructs for therapeutics [130], they offer great advancement opportunities in the field of diagnostics. These developments include the immobilization of aptamers on silicon nitride surfaces [131], functionalizing gold electrodes with bacterial reaction centers [132] and embedding bacteria in 3D constructs [133], all which can be useful in diagnostic applications.

## 4. Conclusions and Outlook

Evolving applications and developments suggest that 3D printing will be a major player in fabricating readily available, cheap, miniaturized, multifunctional and sensitive diagnostic devices. Researchers from different backgrounds have developed diagnostic assays using this versatile technology. 3D printing has been used as a tool for device prototyping and development with photolithography most commonly used because of the availability of materials exhibiting different properties and high resolution. However, its applications now go well beyond prototyping into real-world device fabrication technology. That is, the fully optimized device becomes the final diagnostic tool to be used in hospitals and clinics. In addition, 3D printing is pushing biomedical diagnostic research towards multifunctional devices that can perform several functions, like protein and metabolite extraction and detection using optical and electrochemical signal detection. 

3D printing technology still needs improvement in order to enhance current diagnostic abilities. First, simultaneous printing of multiple materials with high resolution and good compatibility is essential, especially for functional materials like conductive inks and biomimetic substrates. Printing multiple materials with different physical properties would greatly improve the capabilities of 3D printers to produce more complex functional architectures. The ability to print active biomaterials like enzymes and proteins in 3D formats without compromising their basic activity is also an important requirement for better diagnostic devices.

Given the progressive nature of 3D printing, more complex microfluidic architectures can be expected in the near future. Recent research has focused on the development of microfluidic pumps [134], automated flow control valves [135], atomic force microscopes [136] and sophisticated scanning electron microscope sample holders [137]. These are examples of very complex architectures that cannot be readily approached in the averaged bioanalytical laboratory without 3D printers. This illustrates again the significance of incorporating 3D printing in bioanalytical and diagnostic testing research providing a platform for achieving what was believed to be imaginary in the pre-3D printing era.

## Figures and Tables

**Table 1:** Summary of 3D printing techniques. FDM: fused deposition modeling, PLA: polylactic acid, ABS: acrylonitrile butadiene styrene, PC: polycarbonate, DIW: direct ink writing, SLA: stereolithography, MJM: multijet modeling, SLS: selective laser sintering, DLW: direct laser writing.

3D Printing Technique	Principle	Materials	Pros	Cons	Commercially Available Printers
**FDM** [35,36]	Filament Extrusion	PLA, ABS, PC, Acrylates	Inexpensive, Fast, Multiple Materials	Low resolution, Roughness, Leakage	Makerbot, Ultimaker Prusa
**DIW** [46,47,48]	Ink Extrusion	Alginates, Gelatin, Hyaluronates, Epoxy resin	Biocompatible, High Resolution	Extensive Optimization Required	3D-Bioplotter, BioAssemblyBot
**SLA** [54,55]	Light Assisted Polymerization	Acrylates	Good Resolution, Flexibility	Single Material	Form2, FabPro, Nobel
**MJM** [59,64,65]	Printable Photocurable Resin on Support	Multiple Materials	Multiple Materials, High Resolution	Expensive	ProJet, Multijet
**SLS** [67,68]	IR Beam to Sinter Powdered Polymer	Ceramics, Metals, Polymers	Good Resolution, Variety of Substartes	Expensive, Special handling	Fuse 1, Sintratec
**DLW** [73,74]	Two-Photonn Absorption and Polymerization	Polymeric Resin	Exceptional Resolution, Biocompatible	Bulky Instrument	Femtowriter, Tungsten-LAM
